# Impact of Meeting Different Guidelines for Protein Intake on Muscle Mass and Physical Function in Physically Active Older Women

**DOI:** 10.3390/nu10091156

**Published:** 2018-08-24

**Authors:** Andreas Nilsson, Diego Montiel Rojas, Fawzi Kadi

**Affiliations:** School of Health Sciences, Örebro University, 702 81 Örebro, Sweden; diego.montiel@oru.se (D.M.R.); fawzi.kadi@oru.se (F.K.)

**Keywords:** elderly, muscle strength, nutrition, physical activity, physical functioning, Recommended Dietary Allowance (RDA), sarcopenia

## Abstract

The role of dietary protein intake on muscle mass and physical function in older adults is important for the prevention of age-related physical limitations. The aim of the present study was to elucidate links between dietary protein intake and muscle mass and physical function in older women meeting current guidelines of objectively assessed physical activity. In 106 women (65 to 70 years old), protein intake was assessed using a 6-day food record and participants were classified into high and low protein intake groups using two Recommended Dietary Allowance (RDA) thresholds (0.8 g·kg^−1^ bodyweight (BW) and 1.1 g·kg^−1^ BW). Body composition, aerobic fitness, and quadriceps strength were determined using standardized procedures, and self-reported physical function was assessed using the SF-12 Health Survey. Physical activity was assessed by accelerometry and self-report. Women below the 0.8 g·kg^−1^ BW threshold had a lower muscle mass (*p* < 0.05) with no differences in physical function variables. When based on the higher RDA threshold (1.1 g·kg^−1^ BW), in addition to significant differences in muscle mass, women below the higher threshold had a significantly (*p* < 0.05) higher likelihood of having physical limitations. In conclusion, the present study supports the RDA threshold of 0.8 g·kg^−1^ BW of proteins to prevent the loss of muscle mass and emphasizes the importance of the higher RDA threshold of at least 1.1 g·kg^−1^ BW to infer additional benefits on constructs of physical function. Our study also supports the role of protein intake for healthy ageing, even in older adults meeting guidelines for physical activity.

## 1. Introduction

A large body of research has highlighted the role of dietary proteins as primary anabolic stimuli responsible for the maintenance of muscle mass. At an adult population level, a protein intake of 0.8 g·kg^−1^ of bodyweight (BW) represents a recommended daily allowance (RDA) of protein needed for preservation of muscle mass and strength [1,2,3]. However, given the reduced anabolic response to protein ingestion that seems to occur in older adults, an increased protein allowance in the range of 1.0–1.2 g·kg^−1^ BW has been proposed [4]. Indeed, accelerated decline in muscle mass and strength occurs in old adults and is related to impaired physical function, disability, and reduced quality of life [5]. Interestingly, inter-individual variability in the rate of muscle mass wasting and functional decline suggests that lifestyle behaviors, including diet and physical activity, may be key factors for the promotion of health ageing [6]. Therefore, the study of the influence of dietary protein intake on muscle mass and physical function in older adults has received growing attention. It has been shown that a higher protein intake can be associated with preservation of muscle mass [7,8]. For example, a lower protein intake at baseline was associated with a greater loss of lean mass over a 3-year follow-up period in older adults [7]. In contrast, a higher body mass including both fat and lean mass was reported in older women with a protein intake below 0.8 g·kg^−1^ BW [9]. Besides muscle mass, whether protein intake may confer positive effects on functional capacity is important to clarify given the age-related impairment in physical function. Interestingly, higher protein intake has been associated with reduced risk of frailty in older women [10,11,12,13]. Beneficial effects of higher protein intake on physical function, including handgrip strength [8,14,15], the short physical performance battery (SPPB) scores [9], and self-reported physical function [8,15,16,17] have also been reported. However, results from others do not support a significant impact of total protein intake on muscle strength, SPPB scores, and walking speed [18,19,20,21]. These conflicting results may be explained by several factors obscuring the link between dietary protein intake, muscle mass, and physical function. For example, different methods to assess dietary protein intake, including the use of RDA thresholds for classification of high and low intakes, will affect study outcomes. Likewise, which dimension of the physical function and whether objective or self-report assessment methods are used are other relevant factors. In addition, variations in health status among the elderly population must be taken into consideration when evaluating the impact of protein intake on muscle mass and physical function. Differences in study samples regarding the prevalence of diseases and physical impairment likely limit comparability between studies. Noteworthy, as physical function is partly determined by body composition, variations in muscle mass must be considered in order to determine the true impact of protein intake on measures of physical function. Furthermore, physical activity (PA) is a lifestyle behavior with the potential to have a substantial impact on physical function. Indeed, PA has been recognized as an important anabolic stimulus for the regulation of muscle mass [22]. Therefore, when investigating links between protein intake, body composition, and physical function, the influence of PA level should be taken into consideration. However, most previous studies rely on self-reported PA, which is prone to recall bias and less accurate than objective methods [23]. Alongside total weekly amount of PA, accounting for strengthening activities according to PA guidelines for healthy ageing could further clarify the true impact of protein on physical function.

Therefore, the aim of the present study was to elucidate links between dietary protein intake, muscle mass, and objective and self-reported measures of physical function in older community-dwelling women meeting current guidelines of objectively assessed PA.

## 2. Materials and Methods

### 2.1. Subjects

One hundred and twenty-two women aged between 65 and 70 years old were recruited through local advertisement and were subsequently screened for inclusion in the study. To be included in the study, participants had to meet the current guidelines of 150 min per week of moderate-to-vigorous physical activity and be free of diagnosed coronary heart disease, diabetes mellitus, have no disability with respect to mobility, and be non-smokers. A total of 106 women fulfilled inclusion criteria. All procedures were conducted according to standards set by the Declaration of Helsinki. Written informed consent was obtained from all participants and the study was approved by the regional ethics committee of Uppsala (2011/033).

### 2.2. Anthropometry and Body Composition

Body weight (kg) and height (cm) were measured using a digital scale and a portable stadiometer to the nearest 0.1 kg and 0.5 cm, respectively. Subjects having a body mass index (BMI) ≥ 25 kg·m^−2^ were classified as overweight. Skeletal muscle mass index (SMI) was assessed using bioelectrical impedance analysis (BIA) and derived using the equation by Janssen et al. [24].

### 2.3. Assessment of Physical Function

A standardized submaximal exercise on a cycle ergometer (model 874 E; Monark, Varberg, Sweden) was performed during 6 min at a constant workload to assess aerobic fitness [25]. Maximal isometric quadriceps strength was measured using a force sensor (K. TOYO 333A, Toyo-Korea, Seoul, Korea) as previously described [26]. Self-reported physical function limitation was assessed by the 12-item Short Form Health Survey (SF-12) [27]. Participants answered two questions related to the ability to accomplish various daily activities questions on a three-item response scale (limited a lot; limited a little; not limited at all). Participants who reported “limited a little” or “limited a lot” on at least one of the two questions were classified as having a physical limitation, and all other participants were subsequently classified as not having any physical limitation.

### 2.4. Assessment of Adherence to Physical Activity Guidelines

Accelerometer-based assessment of PA during a week was performed using the Actigraph GT3x activity monitor (Actigraph, Pensacola, FL, USA) as previously described [28]. A cut-point of >1324 counts per minute, specifically developed for women over 60 years of age [29], was used to define time spent in moderate-to-vigorous PA (MVPA). Engagement in strengthening activities during the past 12 months was assessed using the EPAQ2 questionnaire [30]. From a list of strengthening activities, such as resistance exercise, participants reported the frequency and average time spent per session. Subsequently, we classified participants in two groups based on whether they engaged in strengthening activities at least twice a week or not.

### 2.5. Dietary Protein Intake

Dietary intake was monitored using a food record over a period of 6 days. Participants were instructed by a nutritionist on how to register their daily food intake with the assistance of a portion size guide developed by the Swedish National Food Agency. Total energy intake and relative macronutrient intakes (E%) were derived using Dietist XP software (Kost och Näringsdata, Bromma, Sweden). Daily protein intake was normalized to bodyweight and expressed as g.kg-1 bodyweight. In accordance with guidelines for protein intake (0.8 g·kg^−1^ BW or 1.1 g·kg^−1^ BW) [1,2,3,4], participants were classified into higher or lower protein intakes. 

### 2.6. Statistics

Data are presented as mean ± standard deviation. All variables were checked for normality and transformed when necessary. Between-group differences in continuous variables were first assessed by using independent samples t-test. Since variations in body composition were related to physical function, adjustment for SMI was performed using analysis of covariance. Binary logistic regression was used to examine the likelihood of having physical limitations in the two protein intake groups based on different RDA thresholds. The model was further adjusted for SMI. A priori statistical power calculations showed that moderate effect sizes were detectable with a power of ≥80% when based on our sample size. Statistical significance was set at *p* < 0.05 and all procedures were performed using SPSS software version 24 (IBM Corporation, New York, NY, USA).

## 3. Results

### 3.1. Characteristics of the Study Population

The mean age, weight, and BMI of the study sample was 67.5 ± 1.8 years, 67.9 ± 11.6 kg, and 25.1 ± 4.0 kg·m^−2^, respectively. The average time spent in MVPA was 67 ± 25 min·day^−1^. Aerobic fitness and isometric quadriceps strength averaged 28.7 ± 7.2 mL O^2^·min^−1^·kg^−1^ BW and 2.7 ± 0.7 N·kg^−1^ BW, respectively. A total of 31.4% reported physical limitations. Compared to women without physical limitations, those reporting limitations had a higher BMI (24.2 ± 3.7 vs. 27.1 ± 3.9; *p* < 0.05), lower SMI (31.4 ± 4.1 vs. 29.2 ± 3.1; *p* < 0.05), lower aerobic fitness (30.1 ± 7.4 vs. 25.2 ± 6.1; *p* < 0.05), and lower isometric quadriceps strength (2.8 ± 0.7 vs. 2.5 ± 0.6; *p* < 0.05). A total of 34% of women reported engagement in strengthening activities at least twice a week and there were no significant differences in body composition variables nor physical function measures between those involved and not involved in this type of activities.

The average energy intake of the study population was 1705 ± 380 kcal·day^−1^, with 46% ± 6%, 34% ± 5%, and 17% ± 2% of the energy derived from carbohydrates, fat, and protein, respectively. There were no significant differences in energy distribution of macronutrients (E%) between groups of protein intakes regardless of whether based on the lower (0.8 g·kg^−1^ BW) or the higher (1.1 g·kg^−1^ BW) protein intake guideline. There were no significant differences in total energy intake or energy distribution of macronutrients (E%) between physically limited and not limited older women.

### 3.2. Influence of Protein Intake on Muscle Mass and Physical Function

The average daily protein intake of the entire sample was 1.03 ± 0.26 g·kg^−1^ BW. When categorizing participants in high and low protein intake based on the lower RDA threshold of 0.8 g·kg^−1^ BW, women in the low protein intake group had a higher BMI and a lower SMI (Table 1) regardless of total energy intake. Similar differences in body composition variables were observed between protein groups based on the higher RDA threshold (1.1 g·kg^−1^ BW). In addition, SMI was significantly lower in those with an intake of <0.8 g·kg^−1^ BW compared to those having an intake of ≥1.1 g·kg^−1^ BW (28.5 ± 3.3 vs. 32.4 ± 3.9, *p* < 0.05).

When comparing objective measures of physical function between protein intake groups, the significant difference in isometric quadriceps strength observed when based on the 1.1 g·kg^−1^ BW threshold (Table 1) became attenuated (*p* = 0.081) after SMI adjustment. No corresponding differences were observed when based on the lower protein intake threshold (0.8 g·kg^−1^ BW).

When based on the lower RDA threshold (0.8 g·kg^−1^ BW), women in the low protein intake group had a higher likelihood of having physical limitations. However, adjustment for SMI attenuated the observed effect (Table 2). Interestingly, when further comparing those below 0.8 g·kg^−1^ BW to those with an intake of ≥1.1 g·kg^−1^ BW exclusively, a significantly higher likelihood of having physical limitations was found in the former group (Odds ratio: 5.48, 95% Confidence interval: 1.34–22.43) even after SMI adjustment. When based on the protein intake threshold of 1.1 g·kg^−1^ BW, those with lower intakes showed a higher likelihood of having physical limitations compared to those meeting this threshold even after SMI adjustment (Table 2).

Finally, we reanalyzed all data adjusted for overweight status (BMI ≥ 25 kg·m^−2^) instead of SMI, which left the results unchanged.

## 4. Discussion

This study highlights the detrimental effects of not meeting the lowest RDA guideline of protein intake on both muscle mass and physical function in elderly women. Importantly, while beneficial effects on muscle mass are obtained by meeting the lower RDA level, meeting the higher RDA intake seems necessary to infer an additional impact on physical function. As all women were physically active, our findings support the role of protein intake for healthy ageing even in older adults meeting guidelines for PA.

Estimation of the daily protein intake necessary to infer beneficial effects on body composition and physical function in the ageing population is a debated question, since the RDA threshold for proteins of 0.8 g·kg^−1^ BW is based on the single endpoint of nitrogen balance [31]. Our findings support the assumption that meeting the RDA intake of 0.8 g·kg^−1^ BW per day is a key factor to prevent the decline in muscle mass in older adults. This is in line with recent data suggesting that reaching this threshold was associated with higher percentage of fat free mass among older men and women [32]. Additionally, a 6-month randomized controlled trial in functionally limited older men indicated that adherence to the RDA of 0.8 g·kg^−1^ BW for protein was sufficient to maintain lean body mass, whereas an intake above 1.3 g·kg^−1^ BW did not infer additional effects on muscle mass or enhance the testosterone-induced anabolic response [33]. While an RDA of 0.8 g·kg^−1^ BW seems to be a protein amount sufficient to prevent sarcopenic loss of muscle mass, our findings indicate that this threshold is insufficient to preserve physical function. Indeed, not meeting an intake of 1.1 g·kg^−1^ BW was associated with a higher likelihood of having physical limitations and lower muscle strength. Conflicting results exist regarding the role and amount of dietary proteins in the maintenance of physical function in older adults. For instance, while ten Haaf et al. failed to demonstrate a relationship between total protein intake, SPPB scores, and handgrip strength in a sample of older community dwelling adults [20], Gregorio et al. reported a significant impact of higher protein intakes on SPPB but not on the physical component of the quality of life questionnaire or handgrip strength [9]. In contrast, lower decreases in handgrip strength were reported in older men and women with higher protein intakes during a 6-year follow-up [14]. In our study, physical function was assessed using both objective measures and self-reported constructs, and women reporting physical limitations had poorer aerobic fitness and strength. Meeting the RDA for proteins was not related to aerobic fitness in our sample of older women, which underlines that protein intake may not be a main factor determining cardiovascular health. Together with data from previous studies, it is suggested that associations between protein intake and physical function are partly dependent on the selected aspect of physical capability.

It is important to note that variations in muscle mass are likely to exert a considerable influence on physical function [34] and it may thus confound the link between protein intake and function. Indeed, associations between protein intake and physical function have been shown to become attenuated following adjustment for body composition [35]. In our study, the association between leg strength and protein intake was attenuated after accounting for variations in muscle mass, which indicates that protein intake may indirectly influence muscle strength through its impact on muscle mass. Importantly, by considering a construct covering a broad range of physical capabilities rather than a single aspect of physical function, our data show that those meeting the higher RDA threshold were less likely to have physical limitations compared to the lower intake group regardless of variation in body composition.

A novel approach used in this study was to exclusively include older women meeting current PA guidelines for good health in order to attenuate the well documented effects of PA on muscle mass and physical function. To further consider the potential influence of PA, we additionally assessed regular engagement in strengthening activities. Based on this approach, our findings support the assumption that protein intake plays an important role in the maintenance of muscle mass and preservation of physical function even in older individuals adhering to health-related PA behaviors. 

Although the dietary record method is a widely accepted standard method for assessing energy intakes, under-reporting is likely to have occurred, which may underestimate true means of total energy intake. Notably, average levels of total energy intake and macronutrient distribution presented in our study were within the range of reference data reported for corresponding age and gender groups [36]. Furthermore, because our sample exclusively comprised apparently healthy and physically active women, our conclusions do not cover groups with more sedentary lifestyles or with overt disease. Of note, skeletal muscle mass was assessed using bioelectrical impedance analysis. In this respect, despite the higher accuracy of other body composition methods including computed tomography or magnetic resonance imaging, bioelectrical impedance analysis is widely used in the diagnosis of sarcopenia. When performed under standardized conditions and using age-specific cross-validated equations, bioelectrical impedance analysis is currently considered to be an accurate measurement of functioning muscle mass in clinical settings and epidemiological studies. Another aspect worth highlighting is that previous studies either did not consider the role of PA or typically relied on crude measures of self-reported PA. In our study, PA levels were objectively assessed and information on specific types of activities relevant for the study outcomes was additionally collected by self-report. Finally, the cross-sectional design of the study prevents us from making inference on causality. Therefore, study outcomes suggesting causal relationships should be interpreted with caution and experimental settings are warranted to determine the true nature of such relationships.

In conclusion, the present study supports the RDA of 0.8 g·kg^−1^ BW of proteins to prevent the loss of muscle mass and physical function in the elderly. Our findings also emphasize that a higher intake of at least 1.1 g·kg^−1^ BW is required to infer additional benefits on constructs of physical function preventing the occurrence of physical limitations in the elderly. Finally, our findings were evident in women who met guidelines for PA, supporting the role of dietary habits in general and protein intake in particular in the promotion of healthy ageing.

## Figures and Tables

**Table 1:** Participant characteristics by groups of protein intake.

	Protein Intake ^c^			
	<0.8	≥0.8	<1.1	≥1.1
*N*	22	84	67	39
Protein intake, g·day^−1^	54.0 ± 9.6	72.4 ± 13.6 *	61.9 ± 10.8	80.2 ± 13.7 *
Relative protein intake, g·kg^−1^ BW·day^−1^	0.71 ± 0.09	1.12 ± 0.23 *	0.87 ± 0.14	1.31 ± 0.18 *
Total energy intake, Kcal·day^−1^	1304 ± 306	1810 ± 324 *	1551 ± 316	1968 ± 336 *
Carbohydrate, % of energy	45.4 ± 6.4	46.2 ± 6.2	46.6 ± 6.1	45.2 ± 6.3
Fat, % of energy	34.0 ± 5.2	34.5 ± 5.3	33.8 ± 5.1	35.3 ± 5.3
Protein, % of energy	17.3 ± 3.5	16.4 ± 2.0	16.5 ± 2.5	16.7 ± 2.1
Body composition				
Weight, kg	76.1 ± 12.6	65.7 ± 10.3 *	71.7 ± 11.5	61.4 ± 8.4 *
Height, cm	164 ± 5	164 ± 6	165 ± 5	164 ± 6
BMI, kg·m^−2^	28.1 ± 4.2	24.3 ± 3.6 *	26.4 ± 4.0	22.9 ± 3.1 *
SMI, % BW	28.5 ± 3.3	31.4 ± 3.9 *	29.9 ± 3.8	32.4 ± 3.9 *
Objective Physical Performance				
VO_2_ max, mlO_2_·kg^−1^ BW·min^−1^ ^a^	27.5 ± 7.9	29.0 ± 7.1	28.0 ± 6.7	29.8 ± 7.9
Isometric Quadriceps Strength, N·kg^−1^ BW	2.5 ± 0.7	2.8 ± 0.7	2.6 ± 0.7	3.0 ± 0.7 *
Self-Reported Physical Limitation, % yes ^b^	55.0	25.6 *	42.9	12.8 *

BW, Body Weight; BMI, Body Mass Index; SMI, Skeletal Muscle Index. ^a^ Based on *n* = 94. ^b^ Based on *n* = 102. ^c^ g·kg^−1^ of BW per day. Data expressed as mean ± standard deviation, unless indicated. * *p* < 0.05.

**Table 2:** Odds ratios (OR, 95% CI) of having a physical limitation for women below the RDA thresholds with those above set as reference.

	Model 1			Model 2		
	OR	95% CI	*p*	OR	95% CI	*p*
Protein intake						
<0.8 g·kg^−1^ BW	3.55	(1.29–9.76)	0.014	2.56	(0.88–7.42)	0.083
<1.1 g·kg^−1^ BW	5.10	(1.76–14.77)	0.003	3.94	(1.31–11.83)	0.015

CI, Coefficient Interval; BW, Body Weight; RDA, Recommended Dietary Allowance. Model 1: unadjusted. Model 2: adjusted for SMI.

## References

[1] Wolfe R.R. (2012). The role of dietary protein in optimizing muscle mass, function and health outcomes in older individuals. Br. J. Nutr..

[2] Baum J.I., Kim I.Y., Wolfe R.R. (2016). Protein consumption and the elderly: What is the optimal level of intake?. Nutrients.

[3] Wolfe R.R., Miller S.L., Miller K.B. (2008). Optimal protein intake in the elderly. Clin. Nutr..

[4] Bauer J., Biolo G., Cederholm T., Cesari M., Cruz-Jentoft A.J., Morley J.E., Phillips S., Sieber C., Stehle P., Teta D. (2013). Evidence-Based Recommendations for Optimal Dietary Protein Intake in Older People: A Position Paper From the PROT-AGE Study Group. J. Am. Med. Dir. Assoc..

[5] Dodds R.M., Roberts H.C., Cooper C., Sayer A.A. (2015). The Epidemiology of Sarcopenia. J. Clin. Densitom..

[6] Nowson C., O’Connell S. (2015). Protein Requirements and Recommendations for Older People: A Review. Nutrients.

[7] Houston D.K., Nicklas B.J., Ding J., Harris T.B., Tylavsky F.A., Newman A.B., Lee J.S., Sahyoun N.R., Visser M., Kritchevsky S.B. (2008). Dietary protein intake is associated with lean mass change in older, community-dwelling adults: The Health, Aging, and Body Composition (Health ABC) Study. Am. J. Clin. Nutr..

[8] Beasley J.M., Wertheim B.C., LaCroix A.Z., Prentice R.L., Neuhouser M.L., Tinker L.F., Kritchevsky S., Shikany J.M., Eaton C., Chen Z. (2013). Biomarker-Calibrated Protein Intake and Physical Function in the Women’s Health Initiative. J. Am. Geriatr. Soc..

[9] Gregorio L., Brindisi J., Kleppinger A., Sullivan R., Mangano K.M., Bihuniak J.D., Kenny A.M., Kerstetter J.E., Insogna K.L. (2014). Adequate dietary protein is associated with better physical performance among post-menopasual women 60–90 years. J. Nutr. Health Aging.

[10] Kobayashi S., Suga H., Sasaki S. (2017). Diet with a combination of high protein and high total antioxidant capacity is strongly associated with low prevalence of frailty among old Japanese women: A multicenter cross-sectional study. Nutr. J..

[11] Nanri H., Yamada Y., Yoshida T., Okabe Y., Nozawa Y., Itoi A., Yoshimura E., Watanabe Y., Yamaguchi M., Yokoyama K. (2018). Sex Difference in the Association Between Protein Intake and Frailty: Assessed Using the Kihon Checklist Indexes Among Older Adults. J. Am. Med. Dir. Assoc..

[12] Lorenzo-López L., Maseda A., De Labra C., Regueiro-Folgueira L., Rodríguez-Villamil J.L., Millán-Calenti J.C. (2017). Nutritional determinants of frailty in older adults: A systematic review. BMC Geriatr..

[13] Beasley J.M., Lacroix A.Z., Neuhouser M.L., Huang Y., Tinker L., Woods N., Michael Y., Curb J.D., Prentice R.L. (2010). Protein Intake and Incident Frailty in the Women’s Health Initiative Observational Study. J. Am. Geriatr. Soc..

[14] McLean R.R., Mangano K.M., Hannan M.T., Kiel D.P., Sahni S. (2016). Dietary Protein Intake Is Protective Against Loss of Grip Strength Among Older Adults in the Framingham Offspring Cohort. J. Gerontol. Ser. A Biol. Sci. Med. Sci..

[15] Granic A., Mendonça N., Sayer A.A., Hill T.R., Davies K., Adamson A., Siervo M., Mathers J.C., Jagger C. (2017). Low protein intake, muscle strength and physical performance in the very old: The Newcastle 85+ Study. Clin. Nutr..

[16] Houston D.K., Tooze J.A., Garcia K., Visser M., Rubin S., Harris T.B., Newman A.B., Kritchevsky S.B. (2017). Protein Intake and Mobility Limitation in Community-Dwelling Older Adults: The Health ABC Study. J. Am. Geriatr. Soc..

[17] Rondanelli M., Klersy C., Terracol G., Talluri J., Maugeri R., Guido D., Faliva M.A., Solerte B.S., Fioravanti M., Lukaski H. (2016). Whey protein, amino acids, and vitamin D supplementation with physical activity increases fat-free mass and strength, functionality, and quality of life and decreases inflammation in sarcopenic elderly. Am. J. Clin. Nutr..

[18] Sahni S., Mangano K.M., Hannan M.T., Kiel D.P., McLean R.R. (2015). Higher Protein Intake Is Associated with Higher Lean Mass and Quadriceps Muscle Strength in Adult Men and Women. J. Nutr..

[19] Scott D., Blizzard L., Fell J., Giles G., Jones G. (2010). Associations Between Dietary Nutrient Intake and Muscle Mass and Strength in Community-Dwelling Older Adults: The Tasmanian Older Adult Cohort Study. J. Am. Geriatr. Soc..

[20] Ten Haaf D., van Dongen E., Nuijten M., Eijsvogels T., de Groot L., Hopman M. (2018). Protein Intake and Distribution in Relation to Physical Functioning and Quality of Life in Community-Dwelling Elderly People: Acknowledging the Role of Physical Activity. Nutrients.

[21] Chan R., Leung J., Woo J., Kwok T. (2014). Associations of dietary protein intake on subsequent decline in muscle mass and physical functions over four years in ambulant older Chinese people. J. Nutr. Health Aging.

[22] Robinson S.M.M., Reginster J.Y.Y., Rizzoli R., Shaw S.C.C., Kanis J.A.A., Bautmans I., Bischoff-Ferrari H., Bruyère O., Cesari M., Dawson-Hughes B. (2018). Does nutrition play a role in the prevention and management of sarcopenia?. Clin. Nutr..

[23] Shad B.J., Wallis G., van Loon L.J.C., Thompson J.L. (2016). Exercise prescription for the older population: The interactions between physical activity, sedentary time, and adequate nutrition in maintaining musculoskeletal health. Maturitas.

[24] Janssen I., Heymsfield S.B., Baumgartner R.N., Ross R. (2000). Estimation of skeletal muscle mass by bioelectrical impedance analysis. J. Appl. Physiol..

[25] Åstrand I. (1960). Aerobic work capacity in men and women with special reference to age. Acta Physiol. Scand. Suppl..

[26] Edholm P., Strandberg E., Kadi F. (2017). Lower limb explosive strength capacity in elderly women: Effects of resistance training and healthy diet. J. Appl. Physiol..

[27] Gandek B., Ware J.E., Aaronson N.K., Apolone G., Bjorner J.B., Brazier J.E., Bullinger M., Kaasa S., Leplege A., Prieto L. (1998). Cross-Validation of Item Selection and Scoring for the SF-12 Health Survey in Nine Countries. J. Clin. Epidemiol..

[28] Nilsson A., Wåhlin-Larsson B., Kadi F., Britta W., Kadi F. (2017). Physical activity and not sedentary time per se influences on clustered metabolic risk in elderly community-dwelling women. PLoS ONE.

[29] Evenson K.R., Wen F., Herring A.H., Di C., LaMonte M.J., Tinker L.F., Lee I.-M., Rillamas-Sun E., LaCroix A.Z., Buchner D.M. (2015). Calibrating physical activity intensity for hip-worn accelerometry in women age 60 to 91 years: The Women’s Health Initiative OPACH Calibration Study. Prev. Med. Rep..

[30] Wareham N.J., Jakes R.W., Rennie K.L., Mitchell J., Hennings S., Day N.E. (2002). Validity and repeatability of the EPIC-Norfolk Physical Activity Questionnaire. Int. J. Epidemiol..

[31] Rand W.M., Pellett P.L., Young V.R. (2003). Meta-analysis of nitrogen balance studies for estimating protein requirements in healthy adults. Am. J. Clin. Nutr..

[32] Beasley J.M., Deierlein A.L., Morland K.B., Granieri E.C., Spark A. (2016). Is meeting the recommended dietary allowance (RDA) for protein related to body composition among older adults? Results from the Cardiovascular Health of Seniors and Built Environment Study. J. Nutr. Health Aging.

[33] Bhasin S., Apovian C.M., Travison T.G., Pencina K., Moore L.L., Huang G., Campbell W.W., Li Z., Howland A.S., Chen R. (2018). Effect of Protein Intake on Lean Body Mass in Functionally Limited Older Men. JAMA Intern. Med..

[34] Bea J.W., Going S.B., Wertheim B.C., Bassford T.L., LaCroix A.Z., Wright N.C., Nicholas J.S., Heymsfield S.B., Chen Z. (2018). Body composition and physical function in the Women’s Health Initiative Observational Study. Prev. Med. Rep..

[35] Isanejad M., Mursu J., Sirola J., Kröger H., Rikkonen T., Tuppurainen M., Erkkilä A.T. (2016). Dietary protein intake is associated with better physical function and muscle strength among elderly women. Br. J. Nutr..

[36] Livsmedelsverket (2012). Riksmaten—Vuxna 2010–2011 Livsmedels-Och Näringsintag Bland Vuxna i Sverige.

